# Treatment of Chronic Diseases of the Heart

**Published:** 1852-10

**Authors:** 


*Treatment of Chronic Diseases of the Heart.*

M. MONNERET has read before the Medical Society of the Hospitals of Paris an essay on the pathology and treatment of chronic diseases of the heart. From the *Revue Medico-Chirurgicale de Paris* we translate for our readers that part of it which relates to treatment; giving, in the first place, a *resume* from the pathology of the writer, in which he maintains that—

The visceral hyperæmias constituting the sole causes of the different morbid conditions observed in diseases of the heart, are themselves produced—1st, by the progress of the valvular lesions and by the cardial hypertrophy; 2nd, by the feebleness of the cardial muscles; 3d, by the primary retardation of the blood in the parenchymæ, from whatever cause; 4th, by general debility of the nervous system. It is to this fourth class of lesions and functional disturbances that we are to direct all our treatment.

The writer then proceeds to point out briefly those diseases having their origin in a mechanical and dynamical alteration of the circulation.

TREATMENT.

1st. *To act upon the heart.*—The physician ought to assure himself of the state of the cardial contraction, and accordingly as it may be too strong or too feeble, he should lessen or increase its power by proper treatment.

2d. *To act upon the capillaries* 1st, by removing more or less directly the congestion of those of the lungs, liver, kidneys, &c.; 2d, by stimulating the systemic capillaries; 3d, by exciting secretion and exhalation, in order to relieve the blood of some of its principles, and thus reduce the hyperæmia.

3d. *To act upon the blood*, either diminishing its stimulant qualities by impoverishing it, or giving to it contrary properties by the aid of excitant medicines which propagate their action throughout the vascular system. Bloodletting, digitalis, stimulants, and still other medicines, have been proposed to meet the indications which

I have pointed out. I shall examine a few of the therapeutical agents most commonly used.

*Bloodletting.* When called to a patient laboring under intense dyspnœa, and presenting all the symptoms of marked bronchial and pulmonary congestion, the first suggestion is to relieve the vascular system, by taking blood. Many physicians obey this impulse, dictated often by the desire of the patient, and the momentary relief which sometimes follows the operation. However, it is not difficult to convince one's self that bleeding, instead of rendering the circulation more easy, only increases the congestions by enfeebling the heart and the capillary vessels of the whole organism. If the operation is repeated, the anxiety and pain of respiration is augmented; the beats of the heart become irregular and tumultuous; the pulse feeble; the blueness and coldness of the surface more intense, and the patient finally succumbs suddenly by syncope, or after a longer agony, full of suffering both for himself and his friends.

Bleeding ought to be still more strongly prohibited where the heart, already enfeebled, becomes dilated and distended with blood, as in passive aneurism of Corvisart. The march of the congestions is more gradual, more slow in active aneurism, but is not arrested by bleeding.

Some physicians of great merit have wished to found the therapeutics of affections of the heart upon the lesions of the valves. Insufficiency of the valves contra-indicates formally the employment of bloodletting, digitalis and debilitants. These agents will, on the contrary, be almost indispensable in contraction. We find here one of those systematic ideas so frequent in medicine. Active and passive aneurism of Corvisart has been succeeded by hypertrophy, and this last in turn, has given place to the valvular theory. We speak and act now only with reference to insufficiency of the valves or contraction, determined or suspected, of the cardiac orifices.

We are then absolutely forced to diagnosticate the anatomical lesion in order to treat the disease. I will remark, in the onset, that these two affections are often found in the same orifice, or that they occupy each one of the two openings. This constitutes the first difficulty. The second consists in the entire absence in many cases of the physical signs of insufficiency of the valves or contraction of the cardiac orifices.

The disturbance of the circulation is sometimes such that it is impossible to distinguish the diagnostic signs in precisely those cases where it is most necessary. Finally, the cardiac hypertrophy and the pulmonary congestions depend, although in rare cases, upon disease of the aorta, of the pericardium, of the pulmonary tissue, of the bronchia, and even of the pleura. Insufficiency of the valves and contraction are in these cases entirely hypothetical, and the practitioner is driven for the fourth time from a source so sterile of therapeutical indications.

On the other hand, of what imporiance is it that valvular disease may be caused by these lesions? They are entirely beyond the sources of art, and cannot serve as a base for therapeutics, as I shall show hereafter. It is not the same with hypertrophy, produced for the purpose of safety and preservation. It retards the sad progress of these congestions, and often even prevents for a long time their development, when it is restricted within certain limits. It is by cardiac hypertrophy that nature struggles against the difficulties of the greater and lesser circulation. It is this also that the practitioner ought not to lose sight of in choosing his remedies to combat the disease. Some diseases, depending upon dilatation of the aortic orifices with insufficiency of the valves, only present very slowly these congestions and dropsical effusions. They are also sometimes less intense and less general than in those subjects in which the cardiac orifices are strongly contracted. But there comes at last a time when these two classes of disease are confounded, and then bloodletting is no longer to be proscribed, or practised upon any other indication than that which is drawn from the dynamic state of the heart and general forces. I consider, finally, as so many contra-indications to bloodletting: First, anæmia, from whatever cause; second, embonpoint, so frequent in persons affected with aneurism, in whom the reaction is very imperfect after depletion; third, old age; fourth, emphysema and difficult expectoration, in which general bloodletting is not advisable. I am convinced that we may replace it in practice by local bleeding, more or less frequently repeated—by the aid of leeches, or of cups upon the regions occupied by the viscera which are the seat of congestion, or over the vessels which are in communication more or less directly with them. Leeches, applied to the extremity of the rectum, act beneficially in overcoming congestion of the liver and the capillaries of the venæ portæ. Congestion of the respiratory organs may also be relieved by scarified cups to the side of the chest and over the sternum. These local and partial depletions are much more efficacious in capillary congestions than general bleeding.

Laennec very truly remarks that, "the observations of practitioners of all ages upon bloodletting, whether general or local, arterial or venous, for the purpose of depletion or derivation, prove a certain independence between the action of the heart and that of the other vessels." I think we have too long neglected to study the effects of local bleeding, which does not enfeeble the organism, and which, nevertheless, diminishes or removes congestion in the same manner as spontaneous and critical hæmorrhages, which, to a certain extent, they resemble.

The debilitating treatment, of which bleeding, low diet, and diluents are the principal agents, has been recommended indiscriminately in all affections of the heart; it is still more used than all others, and yet it exposes the patient, when most enfeebled, to sudden death, as happens by the murderous treatment of Albertini and Valsalva. The better treatment for hypertrophy is that which is founded upon hygiene, especially when called to the patient early, and before there is much obstruction of the capillary circulation.

When congestions have once become established, especially in a slow and chronic manner, and after they have become general, instead of yielding to the debilitating treatment they increase under its influence. Those patients who resist the disease best, and who endure the longest, are vigorous, athletic men, who have been submitted to no treatment, and who often have lived contrary to the precepts laid down by writers upon hygiene. It is useless to enumerate the debilitating agents, they are universally known.

*Digitalis.* There is not a single affection of the heart for which digitalis or digitalline has not been recommended, for the single reason that it reduces the number, and diminishes the intensity of the cardiac contractions. I confess that I have never had great confidence in the action of these medicines, although I have experimented with them with care in a great number of cases. I have become convinced that their use, instead of being general, ought to be restricted to those special conditions which I shall point out, but which rarely occur.

Digitalis is much more dangerous than bloodletting from the fact that the one removes a portion of the natural stimulant of the vessels at the same time that it diminishes their contractile energy, while the other enfeebles the circulating organs, leaving in them the same quantity of fluid to be moved. Digitalis produces, then, an effect directly opposed to the operations of fore-seeing and reparative nature, increasing the size of the heart and tripling as she does its force of contraction. By reducing the functional activity of the heart it only diminishes the *vis a tergo*, and increases the congestion of the parenchymæ.

Digitalis has appeared to me to be especially injurious to patients affected with passive aneurism, and in all cases where the obstacles to the circulation are great and the general forces weak. It should be prohibited still more strongly when the disease has advanced to its later stages, when it is accompanied with all the symptoms of chronic catarrh, œdema of the lungs, and that hyperæmia to which sometimes succeeds pneumorrhagia. The signs which contra-indicate digitalis are feebleness, irregular and intermittent beating of the heart and arteries, and the disappearance of the abnormal sounds previously clear and distinct. It is precisely when digitalis acts in an energetic and prompt manner that we should be fearful of its effects. In such cases I have seen the symptoms of congestion increased, and yield only with difficulty after from five to eight days. In some cases even the propelling force of the heart remains affected till death. I have always present in my mind a case, which I have elsewhere reported, of a patient in which the valves of the aorta were almost entirely detached. The dyspnœa, the anxiety and the disturbance of the circulation constituted an assemblage of alarming symptoms which continued for several days. I administered the digitalis, and the action of the heart was very sensibly reduced; a few days afterwards the patient died by syncope, and with the formation of a well organized fibrinous clot. This case, together with several others less grave in their character, has led me to be very careful in using digitalis. I fear to administer it in all cases in which there is feebleness and irregularity of the cardiac circulation, intense blueness of the surface, sleepiness, drowsiness, and sensible coldness of the extremities. There remains, then, only a small number of cases in which digitalis may be administered with advantage. If, for instance, one is called in time, when the disease is confined to the heart alone, constituting active aneurism, we may associate digitalis with moderate bleeding and especially with a severe dietetic regimen. This treatment has sometimes the effect of immediately arresting the progress of the hypertrophy of the heart, and of diminishing its functional energy. I have not occupied myself with the diuretic properties of digitalis, I will only remark that they are often the true cause of the amelioration in symptoms, which is generally, but falsely, attributed to the special action of the drug. J.

(*To be continued in our next.*)

